# Effect of equalization filters on measurements with kerma‐area product meter in a cardiovascular angiography system

**DOI:** 10.1002/acm2.13444

**Published:** 2021-10-05

**Authors:** Nao Ichikawa, Atsushi Fukuda, Takuma Hayashi, Kosuke Matsubara

**Affiliations:** ^1^ Faculty of Health Science Department of Radiological Technology Kobe Tokiwa University Hyogo Japan; ^2^ Department of Radiological Sciences School of Health Sciences Fukushima Medical University Fukushima Japan; ^3^ Department of Radiation Oncology Shiga General Hospital Shiga Japan; ^4^ Faculty of Health Sciences Department of Quantum Medical Technology Kanazawa University Ishikawa Japan

**Keywords:** equalization filter, kerma‐area product meter, measurement accuracy, percutaneous coronary intervention

## Abstract

**Purpose:**

This study aimed to evaluate the effect of equalization filters (EFs) on the kerma‐area product ($KAP_{Q}^{KM}$) and incident air‐kerma ($K_{a,i,Q}^{KM}$) using a kerma‐area product (KAP) meter. In addition, potential underestimations of the $K_{a,i,Q}^{KM}$ values by EFs were identified.

**Materials and methods:**

A portable flat‐panel detector (FPD) was placed to measure the X‐ray beam area (*A*) and EFs dimension at patient entrance reference point (PERP). Afterward, a 6‐cm^3^ external ionization chamber was placed to measure incident air‐kerma ($K_{a,i,Q}^{ext}$) at PERP instead of the portable FPD. KAP reading and $K_{a,i,Q}^{ext}$ were simultaneously measured at several X‐ray beam qualities with and without EFs. The X‐ray beam quality correction factor by KAP meter ($k_{Q,Q_{0}}^{KM}$) was calculated by *A*, $K_{a,i,Q}^{ext}$ and KAP reading to acquire the $KAP_{Q}^{KM}$ and $K_{a,i,Q}^{KM}$. Upon completion of the measurements, $KAP_{Q}^{KM}$, $K_{a,i,Q}^{KM}$, and $K_{a,i,Q}^{ext}$ were plotted as functions of tube potential, spectral filter, and EFs dimension. Moreover, $K_{a,i,Q}^{KM}/K_{a,i,Q}^{ext}$ values were calculated to evaluate the $K_{a,i,Q}^{KM}$ underestimation.

**Results:**

The $k_{Q,Q_{0}}^{KM}$ values increased with an increase in the X‐ray tube potential and spectral filter, and the maximum $k_{Q,Q_{0}}^{KM}$ was 1.18. $KAP_{Q}^{KM}$ and $K_{a,i,Q}^{KM}$ decreased as functions of EFs dimension, whereas $K_{a,i,Q}^{ext}$ was almost constant. $K_{a,i,Q}^{KM}/K_{a,i,Q}^{ext}$ decreased with an increase in EFs dimension but increased with an increase in tube potential and spectral filter, and the range was 0.55–1.01.

**Conclusions:**

$K_{a,i,Q}^{KM}$ value was up to approximately two times lower than the $K_{a,i,Q}^{ext}$ values by EFs. When using the $K_{a,i,Q}^{KM}$ value, the potential $K_{a,i,Q}^{KM}$ underestimation with EFs should be considered.

## 1 INTRODUCTION

Complex percutaneous coronary intervention (PCI) procedures can result in the administration of high radiation doses to patients. This phenomenon is associated with the risk of radiation‐related skin injuries, such as erythema, epilation, desquamation, and necrosis.^1^, ^2^, ^3^ To prevent these complications, it is imperative to monitor radiation doses in clinical settings.^4^ Accordingly, the International Electrotechnical Commission (IEC) recommends that fluoroscopic equipment must display the kerma‐area product (KAP), cumulative incident air‐kerma (*K_a,i_*), and *K_a,i_* rate (${\dot{K}}_{a,i}$) during procedures.^5^ A KAP meter is commonly preinstalled in fluoroscopic systems for measuring these values.^6^

KAP and *K_a,i_* values are generally used to estimate the individual patient doses or establish diagnostic reference levels that are commonly defined as a percentile of KAP and *K_a,i_* distributions for specific types of procedures in a specific region within a country.^7^, ^8^ Therefore, it is imperative to obtain the accurate KAP and *K_a,i_* values, and the measurement uncertainty for the KAP meter must be within ±35%.^5^, ^9^, ^10^

The coronary arteries run along the surface of the heart, which is surrounded by the lungs. Therefore, image degradation may occur at the edge of the heart by high‐intensity X‐ray beams. Equalization filters (EFs) are X‐ray beam attenuators preinstalled in angiography systems and are used to reduce image degradation by attenuating the high‐intensity X‐ray beams in coronary angiography and PCI procedures.^11^, ^12^, ^13^ EFs mainly attenuate the X‐ray beam at the edge of the X‐ray beam areas and have a lower effect on the central beam axis. Consequently, EFs sharpen the dose gradient of X‐ray beams.

The KAP is theoretically given by the following equation:

(1) $$KAP\;=\int_{A}^{\;}KdA,$$

where *K* is the air‐kerma in the infinitely small X‐ray beam area *dA*, and *A* is the X‐ray beam area.^9^ The definition for KAP measurement is not based on the flatness of the X‐ray beam area. However, *K_a,i_*, which is measured by the KAP meter, is calculated under the assumption that the X‐ray intensity is flat in the X‐ray beam area. The conventional equation to calculate *K_a,i_* is as follows:

(2) $$K_{a,i}=\;\mathrm{KAP}/A,$$

Therefore, we hypothesized that *K_a,i_* values measured by the KAP meter would be underestimated when the EFs sharpen the dose gradient of the X‐ray beam. To the best of our knowledge, no published articles evaluated *K_a,i_* values as a function of EFs dimension. In addition, combining a tube potential with a spectral filter may influence the degree of *K_a,i_* underestimation because X‐ray attenuation using EFs depends on the X‐ray beam quality. This study evaluates the effect of EFs on KAP and *K_a,i_* measured by a KAP meter and identifies the potential underestimation of *K_a,i_* values by the EFs.

## 2 METHODS

### 2.1 Theory

A KAP meter was calibrated at the reference X‐ray beam quality $Q_{0}$, and the KAP values measured by the KAP meter ($KAP_{Q_{0}}^{KM}$) were provided by the following equation:

(3) $$KAP_{Q_{0}}^{KM}=N_{K,Q_{0}}^{KM}\;\;M_{Q_{0}}^{KM}\;k_{TP}^{KM},$$

where $N_{K,Q_{0}}^{KM}$ is the calibration coefficient at the reference X‐ray beam quality $Q_{0}$, $M_{Q_{0}}^{KM}$ is the reading in coulombs of the reference X‐ray beam quality $Q_{0}$, and $k_{TP}^{KM}$ is the temperature and pressure correction factor measured by the KAP meter. The term KM refers to the respective KAP meter measurement. Furthermore, the KAP values at the clinical X‐ray beam quality Q ($KAP_{Q}^{KM}$) are calculated as follows:

(4) $$KAP_{Q}^{KM}=k_{Q,Q_{0}}^{KM}\;\;N_{K,Q_{0}}^{KM}\;M_{Q}^{KM}\;k_{TP}^{KM},$$

where $k_{Q,Q_{0}}^{KM}$ is the X‐ray beam quality correction factor as $N_{K,Q}^{KM}/N_{K,Q_{0}}^{KM}$ and $M_{Q}^{KM}$ is the reading in coulombs of the clinical X‐ray beam quality Q. Accordingly, the KAP values measured by the external ionization chamber at the clinical X‐ray beam quality Q ($KAP_{Q}^{ext}$) are calculated as follows:

(5) $$KAP_{Q}^{ext}=\;A\;k_{Q,Q_{0}}^{ext}\;N_{K,Q_{0}}^{ext}\;M_{Q}^{ext}\;\;k_{TP}^{ext}=\;A\;K_{a,i,Q}^{ext},$$

where A is the X‐ray beam area at the reference point, $k_{Q,Q_{0}}^{ext}$ is the X‐ray beam quality correction factor by external ionization chamber,$\;N_{K,Q_{0}}^{ext}$ is the calibration coefficient, $M_{Q}^{ext}$ is the reading in coulombs at the reference point measured by the external ionization chamber at X‐ray beam quality Q,$\;k_{TP}^{ext}$ is the temperature and pressure correction factor, and $K_{a,i,Q}^{ext}$ is the $K_{a,i}$ at X‐ray beam quality Q. The term ext refers to the external ionization chamber measurement. In theory, $KAP_{Q}^{KM}$ and $KAP_{Q}^{ext}$ values are identical for a uniform X‐ray beam area.

(6) $$\mathrm{KA}P_{Q}^{\mathrm{KM}}=\mathrm{KA}P_{Q}^{\mathrm{ext}},$$

Moreover, the values of $K_{a,i}$ measured by the KAP meter at X‐ray beam quality Q ($K_{a,i,Q}^{KM}$) is expressed as follows:

(7) $$K_{a,i,Q}^{\mathrm{KM}}=\mathrm{KA}P_{Q}^{\mathrm{KM}}\;/A\;=K_{a,i,Q}^{\mathrm{ext}},$$

Using Equations ((4), (5), (6)), $k_{Q,Q_{0}}^{KM}$ is rewritten as follows:

(8) $$\begin{array}{ccc} & & k_{Q,Q_{0}}^{\mathrm{KM}}=\mathrm{KA}P_{Q}^{\mathrm{KM}}\;/N_{K,Q_{0}}^{\mathrm{KM}}\;M_{Q}^{\mathrm{KM}}\;\;k_{\mathrm{TP}}^{\mathrm{KM}}=\mathrm{KA}P_{Q}^{\mathrm{ext}}\;\\ & & /N_{K,Q_{0}}^{\mathrm{KM}}\;M_{Q}^{\mathrm{KM}}\;k_{\mathrm{TP}}^{\mathrm{KM}}, \end{array}$$

### 2.2 Cardiovascular angiography system and instrumentation

**FIGURE 1:**
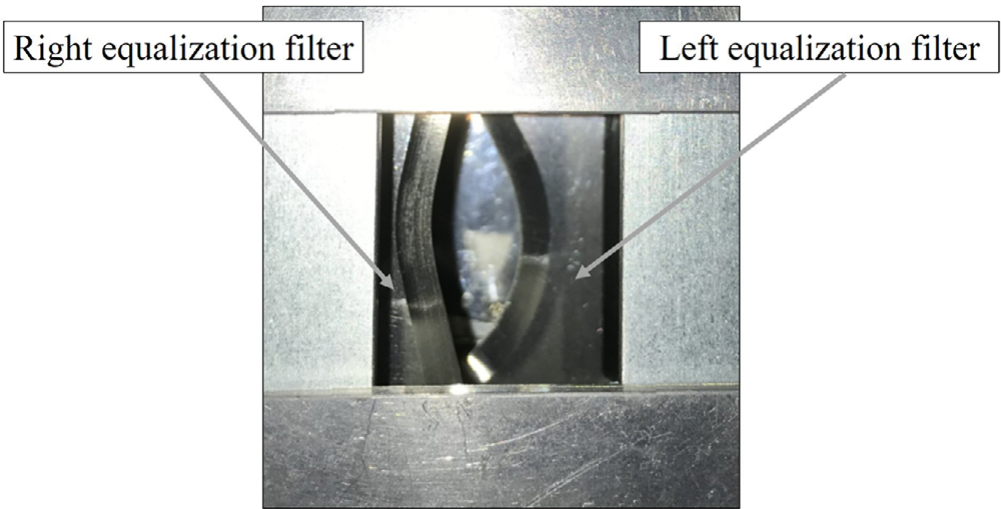
EFs installed in the cardiovascular angiography system. The EFs comprise two filters located in the right and left regions. These filters can rotate and move according to the clinical task. EF, equalization filter

A cardiovascular angiography system (Infinix Celeve‐i, Canon Medical Systems, Nasu, Japan) was employed in this study. This system allows tube potentials from 50 to 125 kV along with spectral filters of 0.2, 0.3, 0.5, and 0.9 mmCu, with field‐of‐view (FOV) size options of 8, 7, 6, 5, and 4.2 inch. The system is equipped with two crescent‐shaped EFs that have tapered made from aluminum (Figure 1). The thickest part of the EFs had 19‐mmAl equivalence at RQR‐5 X‐ray beam quality. A built‐in KAP meter (DIAMENTOR K2S, PTW, Freiburg, Germany) was installed beyond the EFs inside the X‐ray tube assembly to display the KAP value ($N_{K,Q_{0}}^{KM}\;M_{Q}^{KM}$). Furthermore,$\;K_{a,i,Q}^{KM}$ is calculated using Equation (7) because there was no chamber‐in‐chamber installed in the KAP meter. Consequently, the X‐ray beam area at the patient entrance reference point (PERP) is calculated from the source‐to‐PERP distance, source‐to‐image‐receptor distance (SID), and selected FOV on the image receptor.

A 35.6 cm × 43.2 cm portable flat‐panel detector (FPD) (CALNEO Smart C47, Fujifilm, Tokyo, Japan) was used to measure the X‐ray beam area and EFs dimension. To prevent any raw data manipulation, the portable FPD was processed with a fixed mode with a latitude of 4 and a sensitivity of 5.^14^ This mode revealed a log–linear relationship between the *K_a,i_* and signal intensity lower than the saturation of the pixel values.^15^

A 6‐cm^3^ ionization chamber (10 × 6–6, Radcal, Monrovia, CA, USA) calibrated for RQR‐5 X‐ray beam quality was used as an external ionization chamber to measure *K_a,i_* at the central beam axis at the PERP. The $N_{K,Q_{0}}^{ext}$ and $k_{Q,Q_{0}}^{ext}$ values of the external ionization chamber were 0.972 and 1.00, respectively. A dedicated software (Accu‐Gold 2.0, Radcal, Monrovia, CA, USA) was installed on a laptop, and the chamber was connected to the laptop via a digitizer (Accu‐Gold+, Radcal, Monrovia, CA, USA). The software has an automatic $k_{TP}$ correction function, and the temperature and pressure were simultaneously recorded to correct KAP readings.

### 2.3 Measurement of X‐ray beam area and EFs dimension

**FIGURE 2:**
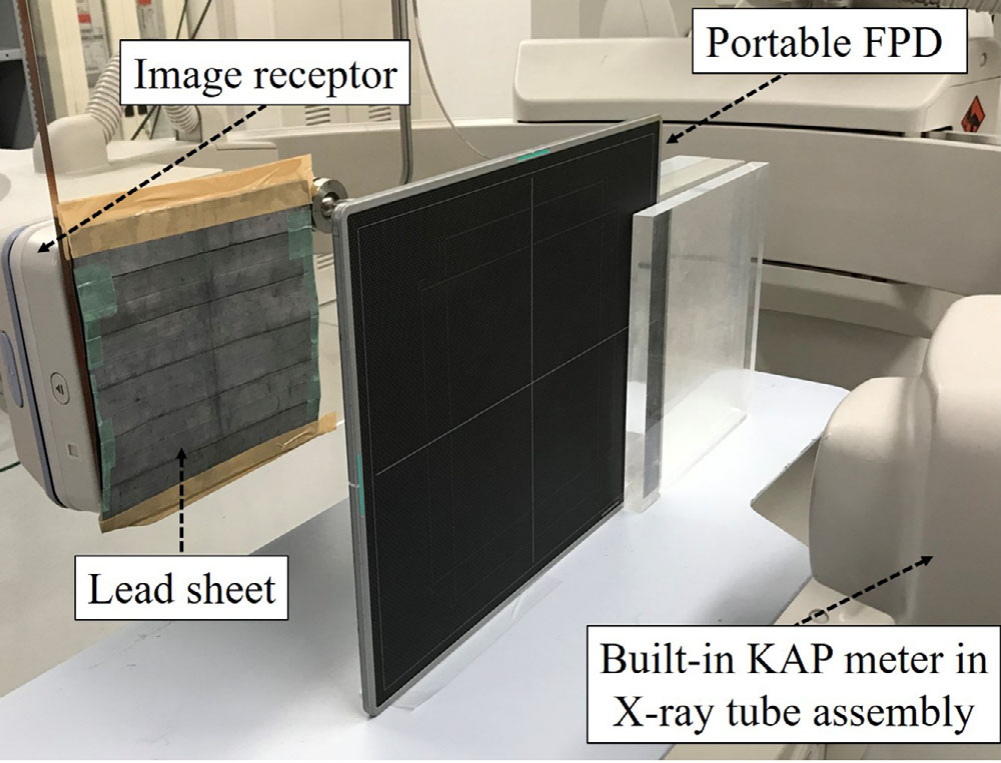
Experimental setup for the measuring X‐ray beam area with a portable FPD. The C‐arm of the cardiovascular angiography system is rotated to the lateral position (90°), and the portable FPD is placed at the PERP. The lead sheet is used to protect the image receptor of the cardiovascular angiography system. KAP, kerma‐area product; FPD, flat‐panel detector; PERP, patient entrance reference point

The X‐ray beam area was required to calculate $KAP_{Q}^{ext}$. Therefore, the portable FPD was placed at the PERP (Figure 2). The C‐arm was rotated to the lateral position (90°), and the SID was set to 100 cm. To protect the image receptor of the cardiovascular angiography system, 2‐mm lead sheets and ceiling pendent‐type 0.5 mm lead‐equivalent protective board were placed in front of the image receptor. The double‐exposure technique was used to identify the full width at half maximum (FWHM), which represents the one‐dimensional X‐ray beam area.^14^, ^15^ The first exposure was performed at tube potential of 70 kV, tube current second of 1 mAs (tube current of 100 mA and pulse width of 10 ms), and FOV of 8 inch to obtain the first density profile. The second exposure at a tube current second of 0.5 mAs (tube current of 50 mA and pulse width of 10 ms) was one‐half of the first exposure, and it was performed to determine the half‐maximum exposure level of the first profile. Finally, the *x*‐ and *y*‐axes FWHMs were measured as distances of the half‐maximum FPD values in the first profile using the ImageJ software (National Institutes of Health, Bethesda, Maryland, USA), and the X‐ray beam area was calculated by multiplying the *x*‐ and *y*‐axes beam widths.^16^

**FIGURE 3:**
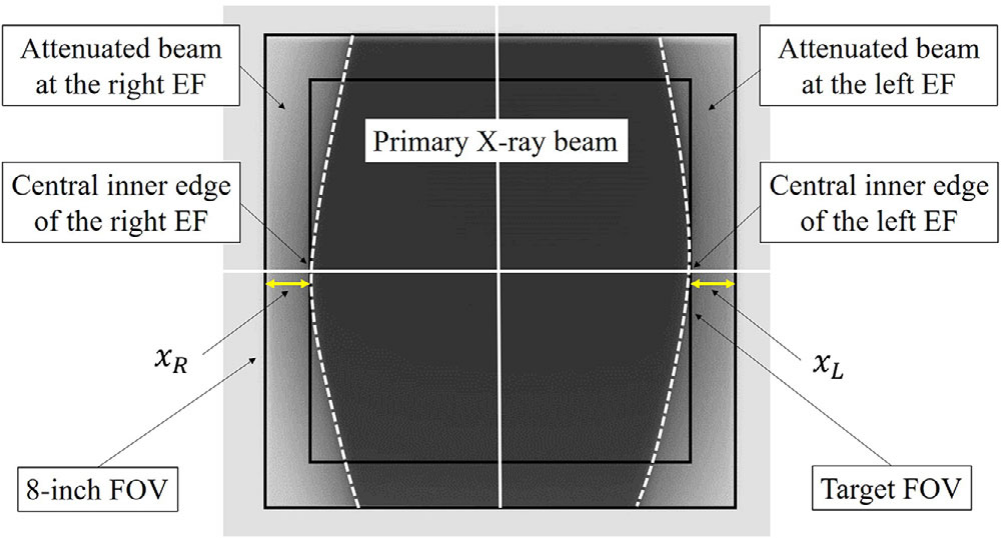
Experimental setup for EFs. To adjust EFs locations, the FOV is first changed to the target size (4.2, 5, 6, 7, or 8 inches). Subsequently, the bilateral EFs are moved so that the central inner edges of the EFs align with the edges of the selected target FOV. After setting the EFs, the FOV is changed to an 8‐inch view. EFs dimension are defined as the lengths from the edges of the X‐ray beam area to right ($x_{R}$) and left ($x_{L}$) inner edges of EFs

The EF dimension was also measured using the identical setting. To adjust the EF dimensions, the FOV was changed to the target size (8, 7, 6, 5, or 4.2 inch) at first. Subsequently, the bilateral EFs were moved so that the central inner edges of the EFs aligned the edges of the target FOV (Figure 3). The FOV was changed to an 8‐inch view before exposure. Upon completion of the exposures, the lengths from the edges of the X‐ray beam area to right ($x_{R}$) and left ($x_{L}$) inner edge of the EFs were measured using the ImageJ software.

### 2.4 Evaluation of $k_{Q,Q_{0}}^{KM}$, $KAP_{Q}^{KM}$,$\;K_{a,i,Q}^{KM}$, and $K_{a,i,Q}^{ext}$

**FIGURE 4:**
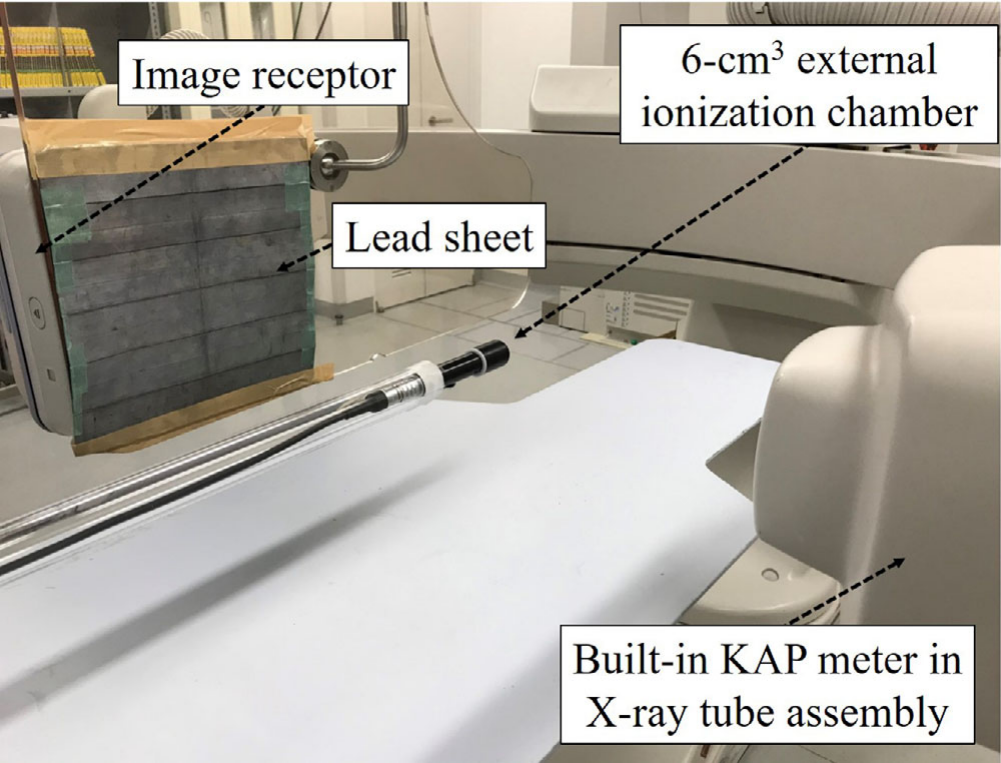
Experimental setup for measuring $K_{a,i,Q}^{ext}$ and KAP reading. The C‐arm of the cardiovascular angiography system is rotated to the lateral position (90°), and the external ionization chamber (6‐cm^3^ ionization chamber) is placed at the PERP. The lead sheet is used to protect the image receptor of the cardiovascular angiography system. $K_{a,i,Q}^{ext}$, incident air kerma at X‐ray beam quality Q measured by external ionization chamber; KAP, kerma‐area product; FPD: flat‐panel detector; PERP, patient entrance reference point

The $k_{Q,Q_{0}}^{KM}$ value is necessary to measure $KAP_{Q}^{KM}$. The geometrical arrangement was similar to that shown in Figure 2, and the external ionization chamber was placed at the PERP instead of the portable FPD (Figure 4). The KAP reading ($N_{K,Q_{0}}^{KM}\;M_{Q}^{KM}$) and $K_{a,i,Q}^{ext}$ were simultaneously measured by the built‐in KAP meter and external ionization chamber, respectively. The X‐ray exposure parameters employed were as follows: tube potentials of 70/100/125 kV, spectral filters of 0.2/0.5/0.9 mmCu, tube current of 200 mA, a pulse width of 10 ms, a frame rate of 15 fps, an exposure of 15 s, an FOV of 8‐inch, and an SID of 100 cm. Upon completion of the measurements, the KAP readings were corrected with the $k_{TP}^{KM}$ measured using Accu‐Gold 2.0 software. Finally, $k_{Q,Q_{0}}^{KM}$ were obtained as a function of the combination of the tube potential and spectral filter using Equation (8).

After calculating the $k_{Q,Q_{0}}^{KM}$, the identical measurements were repeated with EFs. The EFs dimension were identically described in the above section. Upon completion of these measurements, $KAP_{Q}^{KM}$,$\;K_{a,i,Q}^{KM}$, and $K_{a,i,Q}^{ext}$ were plotted as functions of the tube potential, spectral filter, and EFs dimension. Moreover, $K_{a,i,Q}^{KM}$/$K_{a,i,Q}^{ext}$ values were calculated to evaluate the $K_{a,i,Q}^{KM}$ underestimation.

## 3 RESULTS

### 3.1 X‐ray beam area and EFs dimension measurement

**FIGURE 5:**
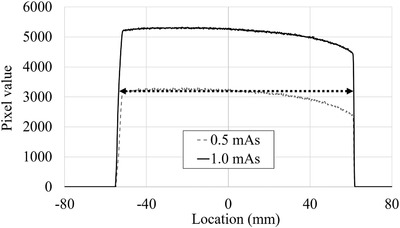
Measurement of *y*‐axis X‐ray beam width with the double‐exposure technique. The double‐exposure technique is used to determine the FWHM. The first exposure at a 70‐kV tube potential and 1‐mAs tube current second is performed to determine the maximum FPD pixel value at the center of the beam. The second exposure is one‐half; it is performed to determine the half‐maximum exposure level of the first profile. Finally, the FWHM is determined as the distance between the half‐maximum FPD pixel values of the first exposure profile. FPD, flat‐panel detector; FWHM, full width at half maximum

Figure 5 demonstrates an example of a *y*‐axis beam width measurement. The half‐maximum FPD pixel value of the first exposure (1 mAs) was the maximum FPD pixel value of the second exposure (0.5 mAs). The FWHMs determined using the double‐exposure technique at the *x*‐ and *y*‐axes were 11.55 and 11.51 cm, respectively. The X‐ray beam area was 132.9 cm^2^.

Similarly, $x_{R}$ and $x_{L}$ measured as the EFs dimension were identical, with values of 0.6, 1.2, 1.8, 2.4, and 2.9 cm, respectively, when the target FOV was changed to 8, 7, 6, 5, and 4.2 inch.

### 3.2 $N_{K,Q_{0}}^{\mathrm{KM}}\;M_{Q}^{\mathrm{KM}}\;k_{TP}^{KM}$, $\;KAP_{Q}^{ext}$, and $k_{Q,Q_{0}}^{KM}$ values as functions of tube potential and spectral filter

Table 1 shows the $N_{K,Q_{0}}^{KM}\;M_{Q}^{KM}\;k_{TP}^{KM}$, $KAP_{Q}^{ext}$, and $k_{Q,Q_{0}}^{KM}$ values as functions of the tube potential and spectral filter. The $N_{K,Q_{0}}^{KM}\;M_{Q}^{KM}\;k_{TP}^{KM}$ and $KAP_{Q}^{ext}$ values increased with an increase in tube potential and decreased with an increase in spectral filter. The $N_{K,Q_{0}}^{KM}\;M_{Q}^{KM}\;k_{TP}^{KM}$ values were identical to the $KAP_{Q}^{ext}$ value at a 70‐kV tube potential and 0.2‐mmCu spectral filter ( $k_{Q,Q_{0}}^{KM}=\;1.00)$, whereas the $k_{Q,Q_{0}}^{KM}$ values increased with increases in tube potential and spectral filter. The maximum $k_{Q,Q_{0}}^{KM}\;$value was 1.18 at a 125‐kV tube potential and 0.9‐mmCu spectral filter.

**TABLE 1:** $N_{K,Q_{0}}^{KM}\;M_{Q}^{KM}\;k_{TP}^{KM}$, $KAP_{Q}^{ext}$, and $k_{Q,Q_{0}}^{KM}$ values as functions of tube potential and spectral filter

Tube potential(kV)	Spectral filter(mmCu)	$N_{K,Q_{0}}^{KM}\;M_{Q}^{KM}\;k_{TP}^{KM}$ (Gy cm^2^)	$KAP_{Q}^{ext}$ (Gy cm^2^)	$k_{Q,Q_{0}}^{KM}$
70	0.2	2.74	2.74	1.00
	0.5	0.98	0.99	1.00
	0.9	0.38	0.38	1.01
100	0.2	6.91	7.24	1.05
	0.5	3.42	3.72	1.09
	0.9	1.83	2.02	1.10
125	0.2	11.27	12.47	1.11
	0.5	6.33	7.35	1.16
	0.9	3.82	4.50	1.18

$N_{K,Q_{0}}^{KM}$, the calibration coefficient at the reference beam quality $Q_{0}$; $M_{Q}^{KM}$, the reading in coulombs of the clinical beam quality Q; $k_{TP}^{KM}$, the temperature and pressure correction factor by kerma‐area product meter; $KAP_{Q}^{ext}$, kerma‐area product by kerma‐area product meter at beam quality Q; $KAP_{Q}^{ext}$, kerma‐area product at beam quality Q calculated by multiplying the X‐ray field with incident air‐kerma measured by external ionization chamber; $k_{Q,Q_{0}}^{KM}$, conversion factor from reference beam quality $Q_{0}$ to beam quality Q.

### 3.3 Evaluation of $KAP_{Q}^{KM}$, $K_{a,i,Q}^{KM}$, and $K_{a,i,Q}^{ext}$ with EFs

**FIGURE 6:**
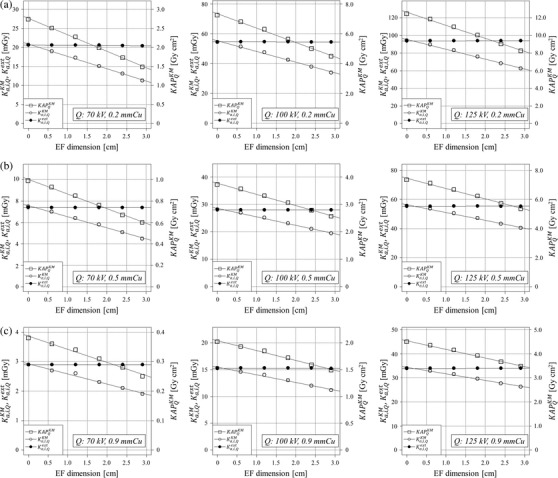
Measurements of $KAP_{Q}^{KM}$,$\;K_{a,i,Q}^{KM}$, and $K_{a,i,Q}^{ext}$. (a) $KAP_{Q}^{\mathrm{KM}}$, $K_{a,i,Q}^{ext}$, and $K_{a,i,Q}^{KM}$ as functions of EFs dimension at 0.2‐mmCu spectral filter. (b) $KAP_{Q}^{\mathrm{KM}}$, $K_{a,i,Q}^{ext}$, and $K_{a,i,Q}^{KM}$ as functions of EFs dimension at 0.5‐mmCu spectral filter. (c) $KAP_{Q}^{\mathrm{KM}}$, $K_{a,i,Q}^{ext}$, and $K_{a,i,Q}^{KM}$ as functions of EFs dimension at 0.9‐mmCu spectral filter

Figure 6a–c shows the obtained $KAP_{Q}^{KM}$, $K_{a,i,Q}^{KM}$, and $K_{a,i,Q}^{ext}$ as functions of tube potential, spectral filter and EFs dimension. $KAP_{Q}^{KM}$, $K_{a,i,Q}^{KM}$, and $K_{a,i,Q}^{ext}$ increased with an increase in tube potential but decreased with an increase in spectral filter. $KAP_{Q}^{KM}$ and $K_{a,i,Q}^{KM}$ also decreased with an increase in EFs dimension regardless of the X‐ray tube potential and spectral filter, whereas $K_{a,i,Q}^{ext}$ values were almost constant.

Table 2 shows the $K_{a,i,Q}^{KM}/K_{a,i,Q}^{ext}$ values as functions of tube potential, spectral filter, and EFs dimension. The $K_{a,i,Q}^{KM}$ values without employing EFs were almost identical with the $K_{a,i,Q}^{ext}$ values at tube potentials of 70, 100, and 125 kV and spectral filters of 0.2, 0.5, and 0.9 mmCu. However, the $K_{a,i,Q}^{KM}/K_{a,i,Q}^{ext}$ values decreased as a function of EFs dimension, and the minimum $K_{a,i,Q}^{KM}/K_{a,i,Q}^{ext}$ was 0.55 at 70‐kV tube potential, 0.2‐mmCu spectral filter, and 2.9‐cm EFs dimension. The decreases in the $K_{a,i,Q}^{KM}/K_{a,i,Q}^{ext}$ values were lower when the high tube potential and high spectral filter were selected.

**TABLE 2:** $K_{a,i,Q}^{KM}/K_{a,i,Q}^{ext}$ values as functions of tube potential, spectral filter, and EFs dimension

		$K_{a,i,Q}^{KM}/K_{a,i,Q}^{ext}$					
		$x_{R}$ and $x_{L}$ (cm)*					
Tube potential(kV)	Spectral filter(mmCu)	0	0.6	1.2	1.8	2.4	2.9
70	0.2	1.01	0.92	0.84	0.73	0.63	0.55
	0.5	1.00	0.94	0.86	0.78	0.68	0.61
	0.9	1.01	0.94	0.88	0.81	0.72	0.66
100	0.2	1.00	0.94	0.87	0.78	0.69	0.62
	0.5	1.00	0.96	0.89	0.82	0.75	0.69
	0.9	1.00	0.96	0.91	0.85	0.78	0.73
125	0.2	1.01	0.95	0.88	0.80	0.72	0.66
	0.5	1.00	0.97	0.91	0.85	0.78	0.73
	0.9	1.00	0.96	0.92	0.87	0.81	0.77

$K_{a,i,Q}^{KM}$, incident air‐kerma measured by the kerma‐area product meter at X‐ray beam quality Q; $K_{a,i,Q}^{ext}$, incident air‐kerma measured by the external ionization chamber at X‐ray beam quality Q; EF, equalization filter; PERP, patient entrance reference point.

* The $x_{R}$ and $x_{L}$ values indicate that the EFs dimension at patient entrance reference point. These values mean the central inner edges of the EFs to the edges of the target FOV distance as shown in Figure 3.

## 4 DISCUSSION

The X‐ray beam area was measured using a portable FPD to calculate $KAP_{Q}^{ext}$. Subsequently, the $k_{Q,Q_{0}}^{KM}\;$values were obtained using Equation (8). The $k_{Q,Q_{0}}^{KM}$ increased from 1.00 (70‐kV tube potential, 0.2‐mmCu spectral filter) to 1.18 (125‐kV tube potential, 0.9‐mmCu spectral filter) with increases in tube potential and spectral filter. These findings underline that the KAP meter has a clear energy dependence. In addition, our results with respect to the $k_{Q,Q_{0}}^{KM}$ values as functions of tube potential and spectral filter were consistent with the results reported by Malusek et al.^17^ The American Association of Physicists in Medicine (AAPM) recommends that the measurement uncertainties in X‐ray dosimeter should be within 10%^10^ and thus $k_{Q,Q_{0}}^{KM}$ should be applied to correct the obtained $KAP_{Q}^{KM}$ values in the clinical settings. However, the X‐ray beam quality can be frequently altered as a function of the employed clinical modes (X‐ray parameters) and geometrical settings, such as working angles or patient physique. To the best of our knowledge, there is currently no automatic correction method in clinical settings.^18^, ^19^, ^20^ Moreover, it is desirable to calibrate the KAP meter using the intermediate X‐ray beam quality used in clinical practice (90–100 kV), as recommended by the AAPM Task Group 190 (TG190).^20^ Although minor geometrical differences between the AAPM TG190 and this study exist, the determination of the KAP correction factor is identical both cases (AAPM TG190 C(KAP) = $k_{Q,Q_{0}}^{KM}$). C(KAP) was 1.05 when employing the AAPM TG190 protocol.

The $K_{a,i,Q}^{KM}$ values were almost identical to the $K_{a,i,Q}^{ext}$ values when the EFs were not employed. However, $KAP_{Q}^{KM}$ and $K_{a,i,Q}^{KM}$ values decreased with an increase in EF dimension at all X‐ray beam qualities, whereas the $K_{a,i,Q}^{ext}$ was almost constant. These results indicated that the $K_{a,i,Q}^{KM}$ values could be significantly underestimated when the EFs sharpened the dose gradient of the X‐ray beam, which was because no chamber‐in‐chamber was installed in the KAP meter. Thus, $K_{a,i,Q}^{KM}$ was calculated using the measured $KAP_{Q}^{KM}$ values and geometrical data in the cardiovascular angiography system. It meant that $K_{a,i,Q}^{KM}/K_{a,i,Q}^{ext}$ values depended on the source‐to‐PERP distance, SID, and the selected FOV. Typically, the FPD was closer to the patient as much as possible to enlarge the imaging area in the clinical setting. Therefore, the X‐ray beam area passing through the EFs increases on the image, and the EFs influence on the $K_{a,i,Q}^{KM}$. The $K_{a,i,Q}^{KM}$ values were conventionally monitored to avoid skin injuries in clinical settings. As mentioned in the results section, the minimum $K_{a,i,Q}^{KM}/K_{a,i,Q}^{ext}$ value was 0.55 when the EF dimension was 2.9 cm at the PERP. The International Commission on Radiological Protection reported that should be kept the dose record if the $K_{a,i,Q}^{KM}$ value exceeded 3 Gy (1 Gy or above for procedures likely to be repeated) for counseling about determination effects.^3^ However, the result of this study suggested that when the $K_{a,i,Q}^{KM}\;$value with EF was 3.0 Gy, the actual value is 5.5 Gy. Moreover, these results underlined that the total uncertainty in the $K_{a,i,Q}^{KM}$ exceeded the most lenient tolerance limit (35%) recommended by the IEC.^5^

The X‐ray beam area was measured using a portable FPD. The method might include a significant error that must be considered in subsequent calculations. The portable FPD could sequentially acquire image data without repositioning, which was advantageous because it could reduce the geometric arrangement error. However, the pixel size of this portable FPD was 0.15 mm, which was larger than the pixel size value of the computed radiography system by 0.1 mm. As a result, the X‐ray beam width might be overestimated by approximately 0.1 mm (0.4%).^14^ Although the measured X‐ray beam area was used to obtain the $KAP_{Q}^{ext}$, this process was not involved in the effects of the X‐ray beam area nonuniformity, such as the heel effect, extra‐focal radiation, and X‐ray energy spectrum, which could affect the $KAP_{Q}^{KM}$. However, these effects can cause errors less than ±3%.^21^

This study has several limitations. First, the $k_{Q,Q_{0}}^{KM}$ values were evaluated with only one built‐in KAP meter in a cardiovascular angiography system. Wunderle et al. showed that $k_{Q,Q_{0}}^{KM}$ in a KAP meter can differ according to the type of device used. Therefore, it is imperative to verify the $k_{Q,Q_{0}}^{KM}$ values for the installed KAP meter before clinical use.^22^ Second, the temperature and pressure values for $k_{TP}^{KM}$ were measured with external ionization chamber. Because the temperature inside the X‐ray tube assembly might increase with an increase in X‐ray production, the $k_{TP}^{KM}$ for the KAP meter might be slightly different from that obtained with the external ionization chamber. Third, the shape, thickness, and material of the EFs might differ among different cardiovascular angiography systems. Consequently, the $K_{a,i,Q}^{KM}/K_{a,i,Q}^{ext}$ values could vary significantly depending on the system used. The $K_{a,i,Q}^{KM}$ values are stored in a digital imaging and communications in medicine (DICOM) radiation dose structured report (RDSR), and information on DICOM RDSR are used for the patient dose management system and skin dose mapping system.^23^ Therefore, the EFs dimension should be in the DICOM tag to correct $K_{a,i,Q}^{KM}$ underestimation in clinical settings. Fourth, the $k_{Q,Q_{0}}^{ext}$ value was set to 1.00 because there were no $k_{Q,Q_{0}}^{ext}$ data for the X‐ray beam quality Q. However, the energy dependence of the external ionization chamber was less than ±2% in the diagnostic energy range,^24^ a value that does not have a significant impact on our findings. Finally, the EF dimension was found to be unrelated to clinical settings. Despite these limitations, we believe that the $K_{a,i,Q}^{KM}$ values can be underestimated when EFs are employed in clinical settings, and this is an issue that a radiation protection supervisor must be aware of.

## 5 CONCLUSION

The $KAP_{Q}^{KM}$ and $K_{a,i,Q}^{KM}$ values decreased as functions of EFs dimension, and the $K_{a,i,Q}^{KM}$ values were underestimated up to 0.55 when the EFs sharpened the dose gradient of the X‐ray beam. Because the $K_{a,i,Q}^{KM}$ values are conventionally monitored to avoid the skin injury in clinical settings, care should be taken so that the actual skin dose may be approximately two times larger than the $K_{a,i,Q}^{KM}$ values. Moreover, the total uncertainty in the $K_{a,i,Q}^{KM}$ values exceeded the tolerance limit recommended by the IEC when EFs were used. Therefore, it is imperative to consider the potential underestimation of $K_{a,i,Q}^{KM}$ when using the EFs in clinical settings.

## AUTHOR CONTRIBUTIONS

Nao Ichikawa: Conception and design of the study, analysis and interpretation of data, collection and assembly of data, drafting of the article, and final approval of the article. Atsushi Fukuda: Conception and design of the study, analysis and interpretation of data, critical revising, and final approval of the article. Takuma Hayashi: Conception and design of the study, analysis and interpretation of data, collection and assembly of data, and final approval of the article. Kosuke Matsubara: Conception and design of the study, analysis and interpretation of data, and final approval of the article.

## CONFLICT OF INTEREST

The authors declare no conflict of interest.

## Supporting information

SUPPORTING INFORMATION

## Data Availability

Data are available on request from the authors.
