# Supplementary material for: Effect of equalization filters on measurements with kerma‐area product meter in a cardiovascular angiography system
Source: J Appl Clin Med Phys. 2021 Oct 5;22(12):177–85. doi: 10.1002/acm2.13444 (PMC8664145; doi:10.1002/acm2.13444)
Supplement: Supplementary file 1 — SUPPORTING INFORMATION [file ACM2-22-177-s001.docx]

Effect of equalization filters on measurements with kerma-area product meter in a cardiovascular angiography system

Nao Ichikawa,^1^ Atsushi Fukuda,^2^ Takuma Hayashi,^3^ Kosuke Matsubara,^4^

*Department,***^1^** *Institution #1*

*Department of Radiological Technology, Faculty of Health Science,*

*Kobe Tokiwa University,*

*Hyogo, Japan*

*Department,***^2^** *Institution #2*

*Department of Radiological Sciences, School of Health Sciences,*

*Fukushima Medical University,*

*Fukushima, Japan*

*Department,***^3^** *Institution #3*

*Department of Radiation Oncology*

*Shiga General Hospital,*

*Shiga, Japan*

*Department,***^4^** *Institution #4*

*Department of Quantum Medical Technology, Faculty of Health Sciences,*

*Kanazawa University,*

*Ishikawa, Japan*

Corresponding author: Nao Ichikawa, MSc

Corresponding author’s full mailing address:

Department of Radiological Technology, Faculty of Health Science,

Kobe Tokiwa University

2-6-2 Otani-cho, Nagata-ku, Kobe, Hyogo 653-0838, Japan

*Tel.: +81-78-611-1821
Fax: no FAX available*

Email: n_a_o523@yahoo.co.jp

Running title: Ichikawa et al.: Effect of EFs on KAP meter

Author Contribution Statement

1. Nao Ichikawa, MSc

Conception and design of the study, Analysis and interpretation of data, Collection and assembly of data, Drafting of the article, and Final approval of the article

1. Atsushi Fukuda, Ph.D

Conception and design of the study, Analysis and interpretation of data, Critical revising, Final approval of the article

1. Takuma Hayashi, RT

Conception and design of the study, Analysis and interpretation of data, Collection and assembly of data, and Final approval of the article

1. Kosuke Matsubara, Ph.D

Conception and design of the study, Analysis and interpretation of data, and Final approval of the article.
